# Response to Imported Case of Marburg Hemorrhagic Fever, the Netherlands

**DOI:** 10.3201/eid1508.090051

**Published:** 2009-08

**Authors:** Aura Timen, Marion P.G. Koopmans, Ann C.T.M. Vossen, Gerard J.J. van Doornum, Stephan Günther, Franchette van den Berkmortel, Kees M. Verduin, Sabine Dittrich, Petra Emmerich, Albert D.M.E. Osterhaus, Jaap T. van Dissel, Roel A. Coutinho

**Affiliations:** National Institute for Public Health and the Environment, Bilthoven, the Netherlands (A. Timen, M.P.G. Koopmans, S. Dittrich, R.A Coutinho); Leiden University Medical Center, Leiden, the Netherlands (M.P.G. Koopmans, A.C.T.M. Vossen, J.T. van Dissel); University Medical Center, Rotterdam, the Netherlands (M.P.G. Koopmans, G.J.J. van Doornum, A.D.M.E. Osterhaus); Bernhard-Nocht-Institute for Tropical Medicine, Hamburg, Germany (S. Gunther, P. Emmerich); Elkerliek Hospital, Helmond, the Netherlands (F. van den Berkmortel, K.M. Verduin); St. P.A.M.M., Veldhoven, the Netherlands (K.M. Verduin); Academic Medical Center, Amsterdam, the Netherlands (R.A. Coutinho); European Centre for Disease Prevention and Control, Stockholm, Sweden (S. Dittrich); 1 On behalf of the national response team.

**Keywords:** Marburg virus diseases, hemorrhagic fever, exposure, contacts, temperature monitoring, filovirus, viruses, perspective

## Abstract

On July 10, 2008, Marburg hemorrhagic fever was confirmed in a Dutch patient who had vacationed recently in Uganda. Exposure most likely occurred in the Python Cave (Maramagambo Forest), which harbors bat species that elsewhere in Africa have been found positive for Marburg virus. A multidisciplinary response team was convened to perform a structured risk assessment, perform risk classification of contacts, issue guidelines for follow-up, provide information, and monitor the crisis response. In total, 130 contacts were identified (66 classified as high risk and 64 as low risk) and monitored for 21 days after their last possible exposure. The case raised questions specific to international travel, postexposure prophylaxis for Marburg virus, and laboratory testing of contacts with fever. We present lessons learned and results of the follow-up serosurvey of contacts and focus on factors that prevented overreaction during an event with a high public health impact.

## Abstract 2

Adventure tourism may bring this disease to Western countries.

In Western countries, Marburg hemorrhagic fever (MHF) is an imported disease with a low risk of occurrence, but it has a high profile in the public mind (*1*) because it can be transmitted from person to person, the course is fatal in up to 80% of cases, and the reservoir is uncertain (*2*,*3*). The infection is caused by the Marburg virus (MARV), an enveloped, nonsegmented, negative-stranded RNA virus belonging, with the Ebola virus, to the family *Filoviridae*. Although the main transmission route is direct contact with blood or other infected body fluids, transmission by droplets and aerosols cannot be ruled out and has been demonstrated in animal models (*4*).

MARV was identified in 1967 in Marburg, Germany, during a laboratory outbreak caused by handling tissues of African green monkeys (*5*,*6*). From 1975 through 1987, sporadic cases occurred in South Africa (1975, when the index case, a person exposed in Zimbabwe, was diagnosed in South Africa) (*7*) and in Kenya (1980, 1987) (*8*–*10*). Outbreaks were reported from the Democratic Republic of Congo in 1998–2000 (*11*,*12*), Angola in 2004–2005 (*2*) and Uganda in 2007 (*13*). Nonhuman primates and bats are suspected as sources of infection, but their role in the natural reservoir for MARV and transmission to humans is unclear (*14*).

In July 2008, an imported case of MHF was diagnosed in the Netherlands. We describe the public health response involving the management of 130 contacts at risk of acquiring the disease.

## The Case

On July 5, 2008, a 41-year-old woman was referred by her general practitioner to the Elkerliek Hospital because of fever (39^°^C) and chills of 3 days’ duration after returning from a June 5–28 holiday in Uganda. She was placed in a hospital room with 3 other patients. Malaria was ruled out by 3 negative blood films. Routine bacteriologic tests were performed, and empiric treatment with ceftriaxone, 2 g/day, was started. On July 7, hemorrhagic fever was included among other infectious causes in the differential diagnosis because of rapid clinical deterioration and impending liver failure. An ambulance stripped of all unnecessary devices and equipped in accordance with strict isolation protocols transferred the patient to a single room with negative air pressure ventilation and anteroom in the Leiden University Medical Centre (LUMC).

After admission, rash, conjunctivitis, diarrhea, liver and kidney failure, and finally, hemorrhaging developed in the patient. Extensive bacteriologic and virologic analyses were conducted, and plasma samples were sent to Dutch national laboratories and to the Bernhard-Nocht-Institute for Tropical Medicine (BNI) in Hamburg, Germany, for testing to detect antibodies to and RNA from filoviruses. Initial laboratory results from the Dutch national reference laboratory were ambiguous for hemorrhagic fever. On July 10, BNI reported a positive reverse transcription–PCR result for MARV (*15*), which was confirmed by sequence analysis of the polymerase gene. The strain was related to, but distinct from, known isolates. MARV was confirmed by PCR by the Department of Virology at Erasmus Medical College (Rotterdam, the Netherlands). On July 11, the patient died of consequences of cerebral edema.

## Travel History and Hypotheses for the Source of Infection

The patient’s travel group consisted of 7 Dutch tourists and 2 guides. Three of the tourists, including the patient, and 1 guide visited an empty cave on June 16 in Fort Portal and the Python Cave in the Maramagambo Forest on June 19. The patient’s partner recalled bats flying around in the latter cave, bumping against the visitors, and large amounts of droppings on the ground. She incurred no bite wounds, and no preexisting wounds were exposed to bats. On July 23, the travel group came within 5 m of gorillas in the wild and visited a village inhabited by pygmies, where they saw an elderly sick woman lying under a blanket.

We postulated that the most probable source of MARV infection was the visit to the Python Cave, known for its colony of Egyptian fruit-eating bats (*Rousettus aegyptiacus*). The party had photographed these bats, and this species of bat has been shown to carry filoviruses, including MARV (*16*,*17*) in other sub-Saharan locations. We estimated the incubation period of the infection to be 13 days.

## Organization of Public Health Response

On July 8, the attending physician at the LUMC notified the Dutch public health authorities about the case. A national outbreak response team was formed of clinicians, medical microbiologists and virologists, public health specialists, staff members from the national response unit, and a press officer. This team convened a nearly daily teleconference to 1) to perform a structured assessment of the public health risks in the 2 hospitals and in the community, 2) perform risk classification of contacts, 3) issue guidelines for follow-up, 4) provide information to professionals and media, and 5) monitor progression of crisis response.

Immediately after the diagnosis was confirmed, on July 10, a press conference was held. Various press statements emphasizing the control measures designed to prevent secondary transmission followed the press conference. The World Health Organization was notified according to the International Health Regulations by the National Focal Point, and international warnings were issued through the Early Warning and Response System and through ProMED.

## Management of Contacts

Although MARV infectivity is highest in the last stage of the disease, when severe bleeding coincides with high viral load, we considered the onset of fever (July 2) as the starting point for contact monitoring. Follow-up measures tailored to the risk group were undertaken during the 21 days after last possible exposure (*14*,*18*,*19*). The high-risk group comprised anyone with unprotected exposure of skin or mucosa to blood or other body fluids of the index patient. It included the other 3 patients in the patient’s room at Elkerliek and personnel who handled her specimens without protection. The low-risk contacts were LUMC and ambulance personnel who had employed the appropriate personal protective measures while caring for the patient or diagnostic samples. Persons who had been near the patient during her holiday, the return flight, and stay in the Netherlands until Elkerliek admission but who were not exposed to her body fluids during her febrile illness and personnel from reference laboratories who worked under BioSafety Level 3 conditions were categorized as casual contacts.

A total of 130 at-risk contacts were identified, 64 at high risk and 66 at low risk (Table). High-risk contacts were required to record their temperature 2×/day, report to the local health authorities 1×/day, and postpone any travel abroad. The low-risk contacts were asked to record their temperature 2×/day and to report to local health authorities if it was >38°C. No limits were imposed on the casual contacts.

**Table:** Control measures targeting contacts with risk for exposure to Marburg virus, the Netherlands, 2008*

Type of contact	Date of exposure, Jul	No. persons	Risk for exposure	Measures		
				Temperature monitoring 2×/day	Daily temperature reporting to health authorities	Asked to limit travel and to not leave the country
Household/family contacts	2–8	4	High	Yes	Yes	Yes
Persons exposed in hospital ward	5–7	6	High	Yes	Yes	Yes
GP of the index case-patient	5	1	High	Yes	Yes	Yes
Healthcare workers, Elkerliek Hospital	7	33	High	Yes	Yes	Yes
Local laboratory workers	5–7	18	High	Yes	Yes	Yes
Ambulance staff	7	2	Low	Yes	No	No
Health care workers, LUMC	7–11	66	Low	Yes	No	No

*GP, general practitioner; LUMC, Leiden University Medical Centre.

Because asymptomatic MARV infection is rare (*20*,*21*) and thus unlikely to play a role in spreading the infection, we restricted further clinical and laboratory evaluation to persons with a temperature >38°C, measured at 2 points 12 hours apart. Every case of fever was to be assessed on an individual basis by the response team. Three academic hospitals provided stand-by isolation facilities for admission of contacts.

On August 1, the temperature monitoring of contacts ended. Fever of at least 12 hours’ duration or clinical signs of MHF did not develop in any of the contacts. Fever within 21 days did not develop in any of the travel companions and local guide who joined the patient in the bat cave. Because sustained fever did not develop in any of the high-risk or low-risk contacts during the surveillance period, no clinical or laboratory follow-up for MARV was needed. The Technical Appendix summarizes other findings during the monitoring period, dilemmas encountered with respect to travel restrictions, postexposure options in case of a high-risk accident, and laboratory diagnosis in the early stage of infection. The Technical Appendix also describes laboratory procedures used.

## Serologic Follow-up

To identify asymptomatic seroconversion, a serosurvey was undertaken of 85/130 (65%) contact persons who participated in the study. They represented 78% (50/64) of high-risk contacts and 53% (35/66) of low-risk contacts and included the Dutch visitors to the bat cave. Blood samples were collected from December 2008 through February 2009, 5–7 months after possible exposure. The laboratory testing was performed at the BNI in Hamburg by using an immunofluorescent antibody (IFA) assay.

The IFA slides were prepared using the MARV strain of the index patient. Details about the laboratory testing are given in the Technical Appendix. In 2 initial evaluations, all but 2 samples were negative for antibodies against MARV. Additional screening found that all serum samples tested negative for immunoglobulin (Ig) G and IgM to MARV.

## Discussion

We have described the public health response to the case of MHF in a Dutch woman returning from travel abroad, who was most likely exposed to MARV by visiting a bat cave. Outbreaks caused by filoviruses constitute a serious public health threat in sub-Saharan countries and have disruptive consequences at the individual and societal level. In countries in which these viruses are not endemic, imported cases occur only sporadically and are associated with little or no secondary transmission (*22*). Our patient represents a rare case of MARV infection imported to a Western country, and her case is unusual in that her only likely exposure was visiting a bat cave while traveling in Uganda. Insectivorous bats may have been the source of sporadic cases in Zimbabwe in 1975 (*23*) and Kenya in 1980 and 1987 (*8*,*9*). Furthermore, epidemiologic evidence linked a large outbreak of MHF in Durba (Democratic Republic of Congo) to a mine containing a large population of fruit-eating bats (*24*). Although the source of infection in our case is not certain, circumstantial evidence points to transmission in the Python Cave. Ecological surveys to assess the presence of infected bats in that cave are ongoing (P. Rollin, pers. comm.).

Our case shows that unnoticed exposure to an unknown reservoir in a country with no apparent cases of MHF can lead to infection. In countries with previous cases of MHF, entry into bat caves should certainly be avoided until we know the role of bats as reservoir for MARV. The importance of MHF for western countries may be increasing, with more persons traveling to high-risk regions and incurring exposure by intrusion into unaccustomed ecological niches. Hospital staff in low-risk countries must be alert to this possibility. In most travelers returning from tropical destinations, fevers are caused by common pathogens or by malaria. However, fever together with rapid clinical deterioration and hemorrhaging in a patient returned from a suspect region should suggest viral hemorrhagic fevers, especially if exposure to a possible reservoir could have occurred.

Inclusion of MHF in the differential diagnosis of a patient triggers an immediate public health response. This response aims primarily at reducing the chance of secondary transmission by identifying contact persons at risk. Person-to-person transmission occurs in countries to which MARV is endemic (*22*) but only once has been reported elsewhere (*23*). In this case we identified 130 contacts with possible risk. Two hospitals, 2 public health departments, and 3 laboratories were involved. We decided to trace all people who were in contact with the index patient after her fever developed and to assess their risk for exposure on a case-by-case basis. All contacts complied with temperature monitoring and daily reporting. All but 2 high-risk contacts postponed further travel until the theoretical incubation period of 21 days had elapsed.

In the Netherlands, statutory power to prevent a healthy person from traveling abroad is limited, but the Public Health Law is being revised, and emergency legal provisions are being considered. Despite various recommendations (*14**,**18**,**25**–**27*), no evidence-based, widely accepted international protocol is available to guide contact classification and monitoring in the case of MHF. Legislation on containment of dangerous pathogens (*1*) and measures applied to contacts differ among countries, sometimes with extreme consequences. These differences, together with privacy issues, make international exchange of information difficult.

The serosurvey of the contacts of this patient confirm that no secondary transmission took place between her and any contact who provided a blood sample. Our results are consistent with those of Borchert et al. (*21*), who found no serologic evidence for asymptomatic or mild MARV infection in a serosurvey of household contacts.

The present case was an exceptional situation in which visiting a tourist attraction led to MHF, a disease with a high potential for overreaction. Given this potential, a rational response must be built on a thorough and evidence-based risk assessment (*1*). The response in the Netherlands was low profile and did not lead to overreacting or public alarm. Its key factors were a coordinated risk assessment and contact monitoring, together with factual updates for health professionals and the public. MHF may be more often encountered in industrialized countries in the future due to adventure travel to regions endemic for MHF.

## Supplementary Material

Technical Appendix Response to Imported Case of Marburg Hemorrhagic Fever, the Netherlands
