# Supplementary material for: Response to Imported Case of Marburg Hemorrhagic Fever, the Netherlands
Source: Emerg Infect Dis. 2009 Aug;15(8):1171–5. doi: 10.3201/eid1508.090051 (PMC2815969; doi:10.3201/eid1508.090051)
Supplement: Technical Appendix — Response to Imported Case of Marburg Hemorrhagic Fever, the Netherlands [file 09-0015_Techapp-s1.pdf]

Response to Imported Case of Marburg Hemorrhagic Fever, the Netherlands

Technical Appendix

Clinical Findings and Dilemmas during Contact Monitoring

Clinical Findings

In 1 high-risk contact, the body temperature once exceeded 38°C, but 12 hours later, the temperature had normalized. Another high-risk contact who shared the patient's room at Elkerliek Hospital was readmitted to that hospital because of heart failure, pulmonary congestion, and subfebrile temperature. His first admission had ended days before his readmission; his condition did not differ between stays and could be attributed to the underlying end-stage heart disease. Several other contacts showed nonspecific symptoms such as nausea and headache, but without fever, and specific follow-up was deemed unnecessary.

The monitoring period led to emotional problems, mostly in high-risk contacts, due mainly to the restrictive measures on daily life and the relatively long period of uncertainty about their prognosis and possible transmission to family members. Psychological support was made available on a case-by-case basis by the occupational health department of the 2 hospitals.

Dilemmas in the Management of Contacts

Problems arose regarding international travel, testing of contacts, and postexposure prophylaxis. By the time Marburg hemorrhagic fever was diagnosed in the index patient, 2 contacts had left for holidays in Italy and the United States, respectively, where they remained for most of the monitoring period. The national authorities of both countries were contacted, and the protocols for temperature monitoring were conveyed with follow-up information on the health status of the 2 persons.

A third contact departed for Poland 3 days before completing the monitoring, after being instructed to carry on the monitoring and stay in daily contact with the Dutch authorities. The Polish authorities were informed because there were doubts about his compliance. Another

person left for Morocco 1 day before the end of the monitoring period, but he kept in touch with the Dutch authorities.

To anticipate possible needlestick accidents or gross breaches of isolation measures by healthcare workers, use of experimental vaccines were assessed in a teleconference with international experts. They favored the vaccine in which attenuated recombinant vesicular stomatitis virus vector expresses the Marburg virus (MARV) glycoprotein (*1–3*) and developed protocols for its use, including regulatory aspects and measures to contain environmental shedding of VSV.

Laboratory Diagnosis in the Early Stage of Infection

Transportation and Processing of Samples

Transport of samples must be organized before sample collection to avoid bottlenecks. We therefore arranged for certified couriers to link hospitals quarantine facilities to laboratories, including the nearest reference laboratory in Germany.

Protocols were designed to encompass essential laboratory testing of severely ill patients, including chemical and bacteriologic diagnostic techniques, biosafety considerations, and methods for decontamination of equipment. No existing preparedness protocols included these considerations. We decided that diagnostic work-ups would be limited to contacts in whom fever developed. In that case, essential equipment for blood chemistry analyses would be placed inside the Intensive Care isolation facility.

Laboratory Assessment of Febrile Contacts: Differential Diagnosis

Protocols were developed for diagnosis of the most probable causes of illness, given the seasonal patters, in which prodromal symptoms resemble those seen in patients with a filovirus infection. These include fever, myalgia, and diarrhoea. Data from physician-based studies of respiratory diseases and gastroenteritis were used as a reference (*4,5*). Contacts with such symptoms would be tested for a range of pathogens to provide an alternative diagnosis. However, their removal from isolation would not be based solely on this testing because common pathogens are often detected in healthy controls.

Filovirus Evaluation in Contact Monitoring

Acute viremia develops in persons infected with Ebola virus, and viral antigens and RNA are detectable in serum, plasma, saliva, and occasionally other secreta (*6,7*). In early stages of infection, results of PCR-based assays have been positive 24–48 earlier than antigen-capture assays, making the PCR the method of choice. Although viral loads in severely ill patients are high, in the early course of illness, viral loads may be barely detectable (*8*). Therefore, proper evaluation of PCR-based methods, with particular emphasis on detection limits, is crucial for reliance on these diagnostics during monitoring. The filovirus diagnostics would therefore be conducted simultaneously in at least 2 laboratories. The Bernhard-Nocht-Institute for Tropical Medicine (BNI) in Hamburg, Germany, provided protocols for PCR-based detection of MARVs. They had been validated in a joint study between P4 laboratories, using all MARV isolates available in these laboratories as reference material (*8*).

Sequence analysis of the patient's MARV strain showed it was most closely related to the first-identified Marburg virus isolate from Uganda, the Popp strain. Therefore, we assumed that detection limits reported for the Popp strain would apply to this strain as well. Reagent kits based on the Panning protocol were assembled at our request and kindly provided within a few days (Thomas Laue; QIAGEN, Hamburg, Germany). Evaluation of this kit, using extracts from patient serum and other possible sample types (throat swab, plasma, serum, feces), provided reliable results. Additionally, strain specific Taqman PCR was designed at the Department of Virology at the Erasmus University Hospital, with detection limits similar to those of the Panning protocol.

Laboratory Procedures Used in the Follow-up Survey

After inactivation and fixation on immunofluorescent antibody assay slides, the samples were stored at –20°C outside the high-containment laboratory, and further investigations using the inactivated virus were performed under BioSafety Level 2 conditions. Testing was performed using 1:10 and 1:40 dilutions in 1× phosphate-buffered saline of the contact sera, with positive (mouse monoclonal antibody against MARV) and negative (MARV-negative mouse sera) controls on every slide. In the initial screening, the presence of immunoglobulin (Ig) G and IgM

was investigated by using IgM and anti-IgG secondary antibodies conjugated with fluorescein isothiocyanate (FITC).

After inconclusive results in the first screening, procedures were repeated using dilutions 1:20, 1:40, 1:80, 1:160, and 1:320 (plus negative and positive control) to enable identification of a potentially higher antibody titer. Inconclusive samples were double-stained with mouse monoclonal antibody and antibodies from the contact sera. The double fluorescence was detected by using 2 differently conjugated secondary antibodies: anti-mouse IgG-rhodamine and anti-human IgG-FITC to differentiate staining between virus particles.

In the initial evaluation of the slides, performed by 2 of the authors (P.E., S.D.), 2 samples could not clearly be identified as negative because they lacked the characteristic round virus inclusions in the cells. However, when virus particles in the infected cells were visualized using monoclonal antibody and overlaying it with the fluorescence of the human antibodies, all activity could be attributed to nonspecific background binding. No overlapping fluorescence of human antibody and mouse monoclonal antibody against Marburg hemorrhagic fever could be observed. Therefore, all tested sera were considered negative for IgG and IgM antibodies to MARV.

References

1. Jones SM, Feldmann H, Stroher U, Geisbert JB, Fernando L, Grolla A, et al. Live attenuated recombinant vaccine protects nonhuman primates against Ebola and Marburg viruses. Nat Med. 2005;11:786–90. PubMed DOI: 10.1038/nm1258

2. Daddario-DiCaprio KM, Geisbert TW, Stroher U, Geisbert JB, Grolla A, Fritz EA, et al. Postexposure protection against Marburg haemorrhagic fever with recombinant vesicular stomatitis virus vectors in non-human primates: an efficacy assessment. Lancet. 2006;367:1399–404. PubMed DOI: 10.1016/S0140-6736(06)68546-2

3. Geisbert TW, Daddario-DiCaprio KM, Williams KJ, Geisbert JB, Leung A, Feldmann F, et al. Recombinant vesicular stomatitis virus vector mediates postexposure protection against Sudan Ebola hemorrhagic fever in nonhuman primates. J Virol. 2008;82:5664–8. PubMed DOI: 10.1128/JVI.00456-08

4. Gageldonk-Lafeber AB, Heijnen ML, Bartelds AI, Peters MF, van der Plas SM, Wilbrink B. A case-control study of acute respiratory tract infection in general practice patients in the Netherlands. Clin Infect Dis. 2005;41:490–7. PubMed DOI: 10.1086/431982

5. de Wit MA, Koopmans MP, Kortbeek LM, Wannet WJ, Vinje J, van Leusden F, et al. Sensor, a population-based cohort study on gastroenteritis in the Netherlands: incidence and etiology. Am J Epidemiol. 2001;154:666–74. PubMed DOI: 10.1093/aje/154.7.666

6. Leroy EM, Baize S, Volchkov VE, Fisher-Hoch SP, Georges-Courbot MC, Lansoud-Soukate J, et al. Human asymptomatic Ebola infection and strong inflammatory response. Lancet. 2000;355:2210–5. PubMed DOI: 10.1016/S0140-6736(00)02405-3

7. Towner JS, Rollin PE, Bausch DG, Sanchez A, Crary SM, Vincent M, et al. Rapid diagnosis of Ebola hemorrhagic fever by reverse transcription-PCR in an outbreak setting and assessment of patient viral load as a predictor of outcome. J Virol. 2004;78:4330–41. PubMed DOI: 10.1128/JVI.78.8.4330-4341.2004

8. Panning M, Laue T, Olschlager S, Eickmann M, Becker S, Raith S, et al. Diagnostic reverse-transcription polymerase chain reaction kit for filoviruses based on the strain collections of all European biosafety level 4 laboratories. J Infect Dis. 2007;196(Suppl 2):S199–204. PubMed DOI: 10.1086/520600
